# Efficacy of a virtual reality-based cognitive interactive training program for children with traumatic brain injuries: study protocol for a parallel-group randomized controlled trial

**DOI:** 10.1186/s13063-024-08049-1

**Published:** 2024-03-13

**Authors:** Jiabin Shen, Yan Wang, Susan Quinn, Stacy J. Suskauer, Julia Birch, Tyler Busch, Adrian Svingos, Roger Crawfis, Keith Owen Yeates, H. Gerry Taylor

**Affiliations:** 1 Department of Psychology, University of Massachusetts Lowell, Lowell, USA; 2 Inpatient Rehabilitation Unit, Spaulding Rehabilitation Hospital, Boston, USA; 3 Kennedy Krieger Institute and Departments of Physical Medicine & Rehabilitation and Pediatrics, Johns Hopkins University School of Medicine, Baltimore, USA; 4 Spaulding Rehabilitation Hospital, Boston, USA; 5 Brain Injury Clinical Research Center, Kennedy Krieger Institute, Departments of Physical Medicine & Rehabilitation, Johns Hopkins University School of Medicine, Baltimore, USA; 6 Department of Computer Science & Engineering, The Ohio State University, Columbus, USA; 7 Department of Psychology, University of Calgary, Calgary, USA; 8 Abigail Research Institute at Nationwide Children’s Hospital, The Ohio State University, Columbus, USA

**Keywords:** Traumatic brain injuries, Virtual reality, Cognitive rehabilitation, Children

## Abstract

**Background:**

Traumatic brain injury (TBI) is a leading cause of disability in children. Cognitive rehabilitation for this population is critical for their long-term health outcomes. This trial aims to evaluate the efficacy of a virtual reality-based program (VICT) for training executive functions in children with TBI.

**Methods:**

A parallel group randomized controlled trial will be conducted among up to 32 children with TBI. Children in the intervention group will receive the VICT training while children in the control group will play a comparable VR game without executive function training. Each participant will be assessed at baseline, post-intervention, and 1-month follow-up. Outcomes will include core executive functions, attention, and health-related quality of life measured by computerized tasks or standardized questionnaires.

**Discussion:**

Cognitive rehabilitation is among the top healthcare needs for pediatric TBI patients. Virtual reality-based training is promising due to its versatile content, flexibility, and potential cost savings for both patients and providers. Findings of this trial will provide data on the efficacy of the VICT program on core executive functions, attention problems, and health-related quality of life and serve as the empirical foundation for future larger multi-site effectiveness trials.

**Trial registration:**

ClinicalTrials.gov NCT04526639. Registered on August 18, 2020.

**Supplementary Information:**

The online version contains supplementary material available at 10.1186/s13063-024-08049-1.

## Administrative information

Note: the numbers in curly brackets in this protocol refer to the SPIRIT checklist item numbers. The order of the items has been modified to group similar items (see http://www.equator-network.org/reporting-guidelines/spirit-2013-statement-defining-standard-protocol-items-for-clinical-trials/).

**Table:** 

Title {1}	Efficacy of a virtual reality-based cognitive interactive training (VICT) program for children with traumatic brain injuries: study protocol for a parallel-group randomized controlled trial.
Trial registration {2a and 2b}.	ClinicalTrials.gov: NCT04526639
Protocol version {3}	Version 5, 11/30/2022
Funding {4}	Eunice Kennedy Shriver National Institute of Child Health and Human Development of the National Institutes of Health under award number R00HD093814
Author details {5a}	Jiabin Shen, Department of Psychology, University of Massachusetts Lowell Yan Wang, Department of Psychology, University of Massachusetts Lowell Susan Quinn, Inpatient Rehabilitation Unit, Spaulding Rehabilitation Hospital Stacy J. Suskauer, Kennedy Krieger Institute and Departments of Physical Medicine & Rehabilitation and Pediatrics, Johns Hopkins University School of Medicine Julia Birch, Spaulding Rehabilitation Hospital Tyler Busch, Brain Injury Clinical Research Center, Kennedy Krieger Institute; Departments of Physical Medicine & Rehabilitation, Johns Hopkins University School of Medicine Adrian Svingos, Brain Injury Clinical Research Center, Kennedy Krieger Institute; Departments of Physical Medicine & Rehabilitation, Johns Hopkins University School of Medicine Roger Crawfis, Department of Computer Science & Engineering, The Ohio State University Keith Owen Yeates, Department of Psychology, University of Calgary H. Gerry Taylor, Abigail Research Institute at Nationwide Children’s Hospital; The Ohio State University
Name and contact information for the trial sponsor {5b}	Yvonne C. Talley. talleyy@mail.nih.gov 301–496-7432
Role of sponsor {5c}	The content is solely the responsibility of the authors and does not necessarily represent the official views of the National Institutes of Health. This funding source had no role in the design of this study and will not have any role during its execution, analyses, interpretation of the data, or decision to submit results.

## Introduction

### Background and rationale {6a}

The Centers for Disease Control and Prevention (CDC) classifies childhood traumatic brain injury (TBI) as the leading cause of death and acquired disability in children, with an estimated 700,000 childhood TBI cases every year in the USA [1–4]. Defined as a disruption in the normal function of a child’s brain that can be caused by a bump, blow, or jolt to the head, or a penetrating head injury, childhood TBIs often result in significant impairment in cognitive functions [1], particularly in executive functions (EFs) due to the vulnerability of the frontal lobes, especially in moderate to severe TBIs [5–7]. Deficits in core EFs (i.e., inhibitory control, working memory, cognitive flexibility) have profound implications for the children’s daily behaviors related to EF [8, 9] (e.g., measured via ecological momentary assessment or EMA) and quality-of-life (QoL) [10], as reflected in increased attention problems [11], poorer academic performance [12], and poorer psychosocial adjustment [13].

Evidence-based EF training programs specifically designed for childhood TBI are unavailable [14–17]. Although a combination of computerized and non-computerized cognitive games has been shown effective in improving healthy children’s EFs [9, 18], *four key obstacles hamper the successful implementation of such interventions in children with TBI: affordability, accessibility, adherence, and generalizability* [19, 20]*.* Virtual reality (VR) offers an exciting alternative EF rehabilitation strategy based on its capability to offer a multitude of activities for training children with TBI in core EFs [8] within a safely controlled, automated virtual environment, which takes minimal physical space and personnel resources to implement in a medical setting. We also expect VR-based EF training to improve adherence due to its technological appeal to the pediatric population.

Despite these potential advantages and the promise of VR in cognitive rehabilitation of adult patients with TBI [21] (in addition to other contexts where VR has been deployed successfully including pain management [22], anxiety reduction [23], and physical rehabilitation in adults with TBI [24, 25]), randomized clinical trials (RCTs) to establish the feasibility, safety, and efficacy of VR-based EF rehabilitation specifically designed for children with TBI remain lacking.

### Objectives {7}

The overall goal of the project is to assess the efficacy of a novel VR-based interactive cognitive training (VICT) program for EF rehabilitation in children with TBI with the following aims:

- Aim 1. Examine VICT’s efficacy in improving core and daily EF skills among children with TBI.
  - *Hypothesis 1.1:* Children in the intervention group will show better performance than controls in trained VR-based EF tasks and untrained NIH Toolbox tasks at post-intervention and follow-up visits.
  - *Hypothesis 1.2:* Children in the intervention group will show better reported daily EF measured by EMA than controls at the follow-up visit.
- Aim 2. Examine VICT’s efficacy in reducing symptoms of inattention among children with TBI.
  - *Hypothesis 2.1: *Children in the intervention group will show fewer symptoms of inattention than controls as measured by the Conners Continuous Performance Test 3rd Edition™ (Conners CPT 3TM) from baseline to the post-intervention and follow-up visits.
  - *Hypothesis 2.2:* Children in the intervention group will show fewer everyday symptoms of inattention than controls on the Behavior Assessment System for Children 3rd Ed (BASC-3) self- and parent-ratings of attention at the follow-up visit.
- Aim 3. Examine VICT’s efficacy in improving HRQOL among children with TBI.
  - *Hypothesis 3.1:* The intervention group will show higher levels of reported HRQOL than controls at follow-up.
  - *Hypothesis 3.2:* The direct effect of the VICT program on HRQOL at follow-up will be mediated by children’s performance-based EF skills and report-based EF-related behaviors and symptoms of inattention at the post-intervention and follow-up visits.

### Trial design {8}

We will conduct a parallel-group randomized controlled superiority trial with active intervention and control groups (1:1 allocation ratio) assessed at baseline (pre-intervention assessment), post-intervention assessment, and 1-month follow-up assessment.

## Methods: participants, interventions, and outcomes

### Study setting {9}

Data will be collected at two clinical settings in the Northeastern region of the USA. The list of study sites can be obtained on clinicaltrials.gov or by emailing the corresponding author of this paper.

### Eligibility criteria {10}

Criteria for study participant eligibility: inclusion criteria—(1) ages 6–17 (inclusive); (2) diagnosed with moderate to severe TBI (determined by multiple factors including but not limited to Glasgow Coma Scale score, additional clinical characteristics, and expert opinions from physician and therapists) within the past 18 months and under 18 years at the time of injury. Patients with positive imaging findings who otherwise would be classified as mild TBI will be considered as moderate TBI; (3) fluent in English; and 3 score < 28 on the Agitated Behavior Scale (if available). Exclusion criteria—(1) comorbidities or premorbid disorders that prevent proper administration of VR and study measures; (2) restriction from using electronic devices; and (3) more than one post-injury seizure activity.

### Who will take informed consent? {26a}

Children and legal guardians will be approached by the site-PI or trained project research assistants approved by IRB only if the children are awake and alert, and if both children and guardians are interested in the study after the initial introduction. The researchers will ensure that guardians and children understand study participation is voluntary and will not affect any treatment in current or future visits. Consent and assent will be obtained only after all questions from the participants are addressed satisfactorily.

### Additional consent provisions for collection and use of participant data and biological specimens {26b}

n/a. There are no additional consent provisions for the collection and use of participant data. No biological specimens will be collected for this project.

## Interventions

### Explanation for the choice of comparators {6b}

The VICT system includes a VR game for the control group that uses the same hardware setup and comparable virtual environment but requires no training of EF skills to play. In this game, children will use the VR hand controller to cast different types of spells (bees, bouncy balls, sparkler spells) on objects in the virtual world. To provide children with an engaging VR gaming experience, spells will have different effects on different objects. This choice of the ‘control group’ game (i.e., comparator) was intended to minimize the possibility of detecting a difference between the intervention and control group that might be due to exposure to VR gaming activities (i.e., if the control group was not asked to play any VR game). The control game will be delivered by trained and masked staff to participants allocated to the control group, with each participant in that group completing one 30-min session of the control game.

### Intervention description {11a}

VICT invites children to rescue an animated character named a “Lubdub” from a heavily guarded castle. The program consists of three challenging and child-friendly tasks that correspond to the three core EFs described earlier. During the game, children are asked to (1) direct a group of sentinels away from the castle gates by indicating the direction of a centrally presented stimulus while inhibiting attention to incongruent directions (VR Task #1 to train Inhibitory Control); (2) successfully open a series of castle gates by replicating the cryptography sequence of items surrounding each gate in forward/backward order (VR Task #2 to train Working Memory); and finally, (3) rescue a Lubdub inside the castle by strategically matching patterns between the Lubdub and the four surrounding guards (VR Task #3 to train Cognitive Flexibility). VICT will be delivered by trained staff to participants allocated to the intervention group, with each participant in that group completing one 30-min session of VICT.

### Criteria for discontinuing or modifying allocated interventions {11b}

The intervention will be discontinued upon participant request. Intervention sessions will be monitored for adverse events and should adverse events occur with the participants, the study staff will immediately discontinue the intervention session and contact the participant’s medical team.

### Strategies to improve adherence to interventions {11c}

Trained staff will explain the intervention procedures to the study participants and will monitor the training session for adherence. Deviations from the protocol will be documented. Staff will meet with the study PI weekly to discuss adherence issues as they arise.

### Relevant concomitant care permitted or prohibited during the trial {11d}

Study participants will be permitted to receive any standard care deemed appropriate by their respective providers without prohibition.

### Provisions for post-trial care {30}

n/a. No ancillary or post-trial care is provided to study participants. No compensation is provided to study participants who suffer harm from trial participation.

### Outcomes {12}

#### Primary outcome measure

1. Changes in performance-based EF scores on the VR-based EF Assessment Task [time frame: baseline (at recruitment/before intervention), post-intervention (after completion of the intervention, up to 2 weeks), follow-up (up to 6 months after completion of the intervention)].

#### Secondary outcome measures

1. Changes in EF scores on the Flanker Inhibitory Control and Attention Test, List Sorting Working Memory Test, and Dimensional Change Card Sort Test from the NIH Toolbox Cognition Battery (NIHTB-CB) [time frame: baseline (at recruitment/before intervention), post-intervention (after completion of the intervention, up to 2 weeks), follow-up (up to 6 months after completion of the intervention)].
2. Report-based EF skills as assessed by *T* scores (*M* = 50, SD = 10) on the BRIEF-2; *T* scores from 60 to 64 are considered mildly elevated, *T* scores from 65 to 69 as potentially clinically elevated, and *T* scores at or above 70 as clinically elevated [time frame: follow-up (up to 6 months after completion of the intervention)].
3. Daily EF skills using the Brief Daily Survey on self-reported EF skills using ecological momentary assessment [time frame: 30 days between post-intervention assessment and follow-up assessment].
4. Attentional problems:
  - Performance-based Conners’ Continuous Performance Test 3rd (CPT 3) [26] [time frame: baseline (at recruitment/before intervention), Post-Intervention (after completion of the intervention, up to 2 weeks), follow-up (up to 6 months after completion of the intervention)];
  - Self/parent-reported Attention Problem Scale of the Behavioral Assessment System for Children 3rd Edition (BASC-3 APS) [27] [time frame: follow-up (up to 6 months after completion of the intervention)].
5. Health-related quality of life using a 23-item PedsQL Generic Core Scales (0–100 after transformation, higher scores indicate better quality of life) [time frame: follow-up (up to 6 months after completion of the intervention)].

#### Other pre-specified outcome measures

1. Motion sickness as assessed by scores on the Simulator Sickness Questionnaire, 0–3, higher scores indicate higher levels of motion sickness [time frame: post-intervention, up to 2 weeks].
2. Perceived exertion as assessed by scores on the Borg Perceived Exertion Scale (6–26, a higher score indicates greater exertion) [time frame: post-intervention, up to 2 weeks].
3. Perceived VR experience as assessed by scores on the VR User Feedback Survey, which provides subjective feedback on the VR intervention (1–5, higher scores indicate better VR experience) [time frame: post-intervention, up to 2 weeks].

### Participant timeline {13}

1. Baseline Assessment: Participants will complete (1) VR and NIHTB-CB tasks; (2) CPT 3; and (3) Child Anxiety Meter (CAM) prior to randomization.
2. Intervention: participants receive either the VR training game or the control game following randomization. The participant will complete VR experience questions about motion sickness, perceived benefits and challenges, and physical exertion after the completion of the intervention.
3. Post-Intervention Assessment: Post-intervention assessment will be scheduled at the time of intervention completion based on family preferences and staff availability, which could be during the same day as the intervention or as soon as possible after the intervention day. Research staff masked to the participant’s group assignment will administer the post-intervention assessment, which consists of the same set of tasks as those in the baseline assessment. Additionally, a brief EMA-EF Survey will be sent to participants to complete daily via a secure Research Electronic Data Capture (REDCap) link or using a paper journal between the post-intervention and follow-up visits.
4. Follow-Up Assessment: Follow-up visits are scheduled at least 1 month following the post-intervention assessment based on family preferences and staff availability. Staff blinded to the participants’ group assignment will administer the following measures: (1) VR and NIHTB-CB tasks; (2) CPT 3; (3) CAM; (4) self-report BRIEF2, BASC-3, and PedsQL; (5) TPVT, and (6) Media Use Survey. A parent will complete the parent-report version of BRIEF-2, BASC-3, and PedsQL.

### Sample size {14}

Power analyses were conducted in G*Power 3.1 to determine the sample size required for the proposed R00 study. We estimated the long-term intervention effect size to be 0.94 (Cohen’s *d*) based on the preliminary data collected from children with moderate/severe TBIs from baseline to follow-up in a pilot study conducted prior to this project [28]. Using a directional one-tail test (as the intervention is expected to improve EFs) and alpha = 0.05, a total sample size of 30 participants is required to offer a power of 0.80. Thus, the study sample size of 32 will provide adequate power to detect the intervention effect.

### Recruitment {15}

A multi-source strategy was developed to maximize the team’s ability to identify eligible patients at the recruitment sites. Researchers will screen for eligible patients through the electronic medical records system on a daily to weekly basis. Study introductions will be conducted via in-person meetings, mailed flyers, emails, and/or phone calls as approved by IRB. Patients may also self-refer from IRB-approved postings on public domains such as clinicaltrials.gov, or study flyers distributed by study collaborators via IRB-approved channels.

## Assignment of interventions: allocation

### Sequence generation {16a}

Random-block randomization will be used for condition allocation at 1:1 ratio with no stratification. Randomization sequence was generated using the *simstudy* package of the R programming language by the project biostatistician. The study team is aware of the use of random block sizes of 2 and 4 in generating the sequence but study team members who are responsible for recruitment do not have access to the actual sequence. Because these team members are unaware of how the block sizes are randomized, we expect minimal bias resulting from this prior knowledge. The intervention assignment was conducted using REDCap based on this generated randomization sequence (by a team member clicking the “randomize” button) without revealing the actual sequence.

### Concealment mechanism {16b}

Randomization will be implemented in the REDCap Randomization Module, which will conceal the allocation until after completion of baseline assessment and will only be available to the RA who conducts the training and is aware of group assignment.

### Implementation {16c}

The randomization sequence will be generated by the project biostatistician and the scheme file will be uploaded to REDCap. Trained staff masked to allocation will enroll participants. A different trained staff member not involved with recruitment or outcome assessment will use the REDCap Randomization module to assign participants (by clicking on a “randomize” button within REDCap) without revealing the entire allocation scheme file.

## Assignment of interventions: blinding

### Who will be blinded {17a}

Study participants, care providers, and outcome assessors will be masked to group assignment. Participant masking will be achieved by exposing only one of the two VR games (training or control) to a participant during the trial. Although during consenting, participants understand there will be two conditions but participants will not be aware of the detailed description of the game in respective conditions. Care providers will be masked by not being involved in any processes after the randomization such as intervention delivery or outcome assessment. Outcome assessors will be comprised of a team of staff different from those who deliver the intervention, thus masked for the interventional condition.

### Procedure for unblinding if needed {17b}

n/a. Unmasking will not be available to study participants, care provider, or outcome assessor during the trial.

## Data collection and management

### Plans for assessment and collection of outcomes {18a}

#### Primary outcome

*Changes in performance-based EF*: This outcome will be measured by scores on the VR-based EF Assessment Task generated by the VICT program for all study participants. This assessment will be performed at the first/baseline study visit before randomization occurs, immediately (up to 2 weeks) after the completion of the intervention, and at the follow-up visit (at least 1 month after the completion of post-intervention assessment). This measure was chosen because of its closeness to the interventional training content so that the study can examine the program’s efficacy in improving trained skills as its primary outcome. This measure has been developed, validated, and applied to pediatric TBI patients using the same procedure in previous research [29].

#### Secondary outcome measures

1. *Changes in Performance-based EF tasks*: This task will consist of three tests described earlier from the NIH Toolbox Cognition Battery administered to all study participants at the first/baseline study visit before randomization occurs, immediately (up to 2 weeks) after the completion of the intervention, and at the follow-up visit (at least 1 month after the completion of post-intervention assessment). This test battery NIH Toolbox was selected because of its wide application in rehabilitation settings by neuropsychologists to examine cognitive (including executive) functions in children with TBI. Acceptable psychometric properties of Toolbox tests have been widely published in related literature [30].
2. *Reporter-based assessment of EF skills*: Both caregiver and self-report versions of the BRIEF-2 will be completed. The BRIEF-2 will be administered at the follow-up visit (at least 1 month after the completion of post-intervention assessment). Acceptable psychometric properties of this measure have been published previously [31].
3. *Daily EF skills*: The Brief Daily Survey is a 5-item self-report of EF skills using ecological momentary assessment methods. This assessment will be performed daily between day 1 and day 30 after the completion of the intervention. As this is a newly developed measure, its psychometric properties have not been established; it was created to examine the daily fluctuations of self-perceived EF following the intervention.
4. *Symptoms of Inattention*: The Conners’ Continuous Performance Test 3rd (CPT 3) [26] will be administered at the first/baseline study visit before randomization occurs, immediately (up to 2 weeks) after the completion of the intervention, and at the follow-up visit (at least 1 month after the completion of post-intervention assessment). The reliability and validity of the CPT3 has been established in previous literature [32]. The self- and parent-reported versions of the Attention Problem Scale of the Behavioral Assessment System for Children 3rd Edition (BASC-3 APS) [27] will also be administered, but only at the follow-up visit (at least 1 month after completion of post-intervention assessment). Acceptable reliability and validity of the BASC-3 has been established in previous literature among pediatric populations [33]. Both instruments were chosen due to their wide application in neuropsychology for attentional assessment.
5. *Health-related quality of life*: The 23-item PedsQL Generic Core Scales (0–100 after transformation, higher scores indicate better quality of life) will be administered at the follow-up visit (at least 1 month after the completion of post-intervention assessment). Acceptable psychometric properties of this measure, selected to assess a more general potential benefit of VR for post-TBI health status, have been previously documented [34].

#### Other pre-specified outcome measures

1. *Motion sickness*: The Simulator Sickness Questionnaire assesses self-perceptions of motor sickness on a 0–3 scale, with higher scores indicating higher levels of motion sickness. This measure will be administered immediately (up to 2 weeks) after the completion of the intervention. The measure has established psychometric properties [35] and was selected to provide information on the subjective experience of participants during the VR intervention.
2. *Perceived exertion*: The single-item Borg Perceived Exertion Scale (6–26, a higher score indicates greater exertion) will be administered immediately (up to 2 weeks) after the completion of the intervention. This measure was selected to assess the physical fatigue of participants during the VR intervention and also has established psychometric properties [36].
3. *Perceived VR experience*: The VR User Feedback Survey provides subjective feedback on the VR intervention (1–5, higher scores indicate better VR experience). This assessment will be administered immediately (up to 2 weeks) after the completion of the intervention. Although this measure has been used in previous pediatric TBI research its psychometric properties are unknown [28].

### Plans to promote participant retention and complete follow-up {18b}

We anticipate that a small proportion of participants might be unable to complete all study visits. The following strategy will be applied to minimize attrition. First, researchers will ensure participants’ understanding of the longitudinal nature of the study during consenting. Second, reminders will be delivered both at the end of a prior visit and before the visit day via emails, phone calls, and/or texting using IRB-approved scripts.

### Data management {19}

All data will be obtained for research purposes only. Research data in this study will include standardized questionnaires, neuropsychological assessment measures, performance data from the VR games, rating scales to assess children’s virtual reality experience, executive functions, attention problems, and quality of life reported by children and/or parents depending on the nature of the measure. All paper-based source files will be securely stored in locked cabinets designated for this study by respective site-PIs and only accessible to IRB-approved research staff. All data from these paper-based research materials as well as those collected in electronic format will first be de-identified before being coded and stored in secure cloud services approved by respective recruitment sites, which will then become accessible by the PI at the University of Massachusetts Lowell. All research data will use a unique study ID for each participant, and only the research team approved by the IRB will have access to the master file that links research data to individually identifiable private information about the participants in the form of a password-protected electronic database stored in secure computing devices at respective recruitment sites. Data quality will be checked by trained research staff supervised by site-PIs and PI for important characteristics such as value range and data type.

### Confidentiality {27}

The confidentiality of study participants will be maintained by assigning each participant a unique study ID number. IRB-approved study personnel at each site will maintain a separate master file that links the participants’ names and their study ID numbers in a password-protected electronic database. The Information Technology Unit at the University of Massachusetts Lowell will store and maintain all de-identified data collected from the study on a secured electronic database (REDCap). All measurement and randomization procedures conducted during the study will only use the study ID number for identification purposes without participants’ names or any other Personal Health Information attached. All study materials will only be accessible to study staff approved by the IRB. Data regarding the participants’ injuries will be derived from their medical records and also included in the secure electronic databases without attachment to any PHI. Study results from the data analysis will be reported only in an aggregated format without any identifying information during and after the trial.

### Plans for collection, laboratory evaluation, and storage of biological specimens for genetic or molecular analysis in this trial/future use {33}

n/a. No biological specimens will be collected.

## Statistical methods

### Statistical methods for primary and secondary outcomes {20a}

Intent-to-treat analysis will evaluate intervention efficacy, controlling for any of the following baseline factors that are significantly different between groups: baseline outcome scores, age, sex, injury severity, recency of injury, verbal intelligence (measured by NIH Toolbox Picture Vocabulary Test), anxiety (measured by CAM), and prior VR gaming experience (measured by Media Use Survey). Specifically, changes in EFs- (primary outcome) will be tested using multiple linear regression (MLR) models with post-intervention/ follow-up scores on VR-based EF tasks and NIHTB-CB tasks or BRIEF-2 rating scores at follow-up as respective dependent variables (DVs). Condition (intervention or control) will be the independent variable (IV) for all models. Changes in daily EF as measured by the EMA approach will be tested using a linear latent growth curve modeling which the growth trajectories of daily EF are estimated across patients as latent variables based on the EMA-EF scores and the condition serves as a predictor of the growth trajectories. Specifically, assuming a linear growth trajectory, each patient is allowed to have a unique initial level (i.e., intercept) and rate of change (i.e., slope) in daily EF across the 30-day period. The average initial level and rate of change as well as variations across patients will be estimated. The initial level and rate of change will be regressed on the condition to test whether the condition impacts patients’ growth trajectories. The EMA-EF scores will be the DV, allowing each patient to have a unique initial level and rate of change in daily EF across the 30-day period. Furthermore, changes in symptoms of inattention (secondary outcome) will be tested using MLR modeling with post-intervention/follow-up scores on CPT 3 and BASC-3 APS scores at follow-up as respective DVs, and condition as the IV. Finally, changes in health-related quality of life (secondary outcome) will be tested using MLR modeling with PedsQL scores at follow-up as DVs and the condition as the IV. Potential differences between the two sites will be evaluated by including the site variable in all the models above as a covariate. In addition, site-by-condition interaction will be included to examine if the intervention effect differs by site.

### Interim analyses {21b}

n/a. No interim analyses were planned.

### Methods for additional analyses (e.g., subgroup analyses) {20b}

n/a. No additional analyses were planned.

### Methods in analysis to handle protocol non-adherence and any statistical methods to handle missing data {20c}

Intent-to-treat analysis will be used to handle non-adherence. If missing data occur in more than 5% of all data points, advanced missing data management techniques (e.g., multiple imputations) will be applied.

### Plans to give access to the full protocol, participant-level data, and statistical code {31c}

Public access to the full protocol and statistical code is available upon reasonable request made to the corresponding author. De-identified participant-level dataset will be deposited and made available to the public through NICHD Data and Specimen Hub (DASH). DASH has policies and procedures in place that are fully consistent with the NIH Data Sharing Policies and applicable laws and regulations. The final dataset will include demographic and clinical data associated with the nature and severity of patients' injuries, self/parent-reported psychological and behavioral data, and performance data on cognitive outcomes. Submitted data will be confirmed with relevant data and terminology standards as well as policies at NIH, NICHD, and DASH.

## Oversight and monitoring

### Composition of the coordinating center and trial steering committee {5d}

The project is coordinated and monitored by the PI and two site-PIs at respective recruitment sites, and each site will be additionally supported by a project coordinator and at least one additional research assistant. The site-PIs are responsible for providing supervision and organizational support to the day-to-day running of the trial. Additionally, the PI has a standing weekly meeting with teams at both sites to address any issues that come up. There is no coordinating center or trial steering committee for this project.

### Composition of the data monitoring committee, its role and reporting structure {21a}

A data monitoring committee (DMC) was determined not to be needed because this study involves only the use of electronic video games as the sole interventional strategy and the risk of this study is expected to be minimal. The PI and site-PIs will assure that informed consent/assent is obtained before enrolling any child, that all participants meet eligibility criteria, and that the study is conducted according to the IRB-approved research plan. Study data will be accessible at all times for the PI and site-PIs to review. The PI and site-PIs will review study conduct (e.g., accrual, drop-outs, protocol deviations) weekly. The PI and site-PIs will review adverse events (AEs) and serious adverse events (SAEs), as well as participants’ discomfort, including fatigue and simulator sickness, in real-time and in aggregate weekly. The PI and site-PIs will ensure that all protocol deviations, AEs, and SAEs are reported to the sponsor and IRB according to the applicable regulatory requirements.

### Adverse event reporting and harms {22}

Trained study staff will approach patients and their families in a sensitive and caring fashion to minimize any potential psychological uneasiness in participating in this study. Over the entire study period, the study staff will receive extensive training in identifying any significant physical or mental discomfort. Breaks during both the VR training sessions and various outcome measures will be available to participants upon request. Participants who feel uncomfortable either physically or psychologically using the VR or any of the study measures will have the option to withdraw from the study at any time without affecting their access to standard care. Should adverse events occur with the participants, the study staff will immediately terminate the VR game and contact attending doctors/nurses for assessment. Adverse events will be monitored in real time and reported to the corresponding institutional IRB.

### Frequency and plans for auditing trial conduct {23}

Trial conduct will be audited on an annual basis by the Mass General Brigham Human Research Office/Institutional Review Board, which holds the single IRB framework for this study. Trial conduct will be monitored weekly by site-PIs and discussed in standing weekly meetings with the PI.

### Plans for communicating important protocol amendments to relevant parties (e.g. trial participants, ethical committees) {25}

Important protocol amendments will be submitted to institutional ethical review committees for review and approval before changes are implemented and will follow IRB committee guidelines with respect to the need for informing study participants of these changes. Once approved, such changes will also be updated in the registration records at clinicaltrials.gov for review and public release.

### Dissemination plans {31a}

1. Peer-reviewed journal articles

We expect to publish 1–2 peer-reviewed papers in the field of pediatric trauma research arising from this project. The PI will oversee all aspects of research design, data collection, data analyses, and manuscript preparation in collaboration with the research team.

2. National conference presentations

We also plan to submit abstracts to the national professional conferences to reach a broad audience in the field of rehabilitation medicine and pediatric behavioral sciences.

3. Dissemination among clinical, patient, and public communities

We will use the following strategies to communicate the study findings using plain-language summaries from this project to a broader audience, including (i) media outreach and creation of related materials, (ii) professional networks, and (iii) key professional organizations. Marketing may include the utilization of infographics and press releases. We will collaborate with the institutional marketing team on the development and execution of the dissemination plan. The main study findings will also be updated on the clinicaltrials.gov record for public access.

## Discussion

Consistent with the promising safety and feasibility data from the pilot studies, the VR training program and the trial protocol have been largely working as expected since the patient recruitment began in 2021 at Spaulding Rehabilitation Hospital (SRH). The study team did face an operational challenge—slower than planned recruitment pace, which is partly due to the relative rarity of the target medical diagnosis (a common challenge for many small-scale pediatric TBI intervention studies), despite the research team’s extensive efforts to screen past, current, and incoming patients at the site. This recruitment challenge was further worsened by the lower-than-usual patient volume and staff shortage persisted through the pandemic and post-pandemic period. To address this challenge, two lines of efforts have been implemented. First, the SRH team utilized the strength of the Spaulding Rehabilitation Network and the Mass General Brigham (MGB) System to query the MGB Research Patient Data Registry (RPDR) and inform other healthcare providers who may encounter potential participants about the study. Second, upon discussion with the sponsor and the SRH team, the project received support from a second recruitment site—Kennedy Krieger Institute (KKI; Baltimore, MD) with training and IRB review started in 2022 and participant enrollment since summer 2023.

## Trial status 

Protocol Version 5 (11/30/2022). Recruitment began on September 3, 2021, with completion expected on August 31, 2024.

### Supplementary Information

**Additional file 1.** SPIRIT Figure.

## Data Availability

The de-identified final trial dataset will be available for access by JS. De-identified participant-level dataset will be deposited and made available to the public through NICHD Data and Specimen Hub (DASH) within 1 year after publication of the main research findings from this trial.

## Abbreviations

EF: Executive function
VR: Virtual reality
TBI: Traumatic brain injury
VICT: Virtual reality-based interactive cognitive training
