# Supplementary figures and images for: Efficacy of a virtual reality-based cognitive interactive training program for children with traumatic brain injuries: study protocol for a parallel-group randomized controlled trial
Source: Trials. 2024 Mar 13;25:185. doi: 10.1186/s13063-024-08049-1 (PMC10935958; doi:10.1186/s13063-024-08049-1)

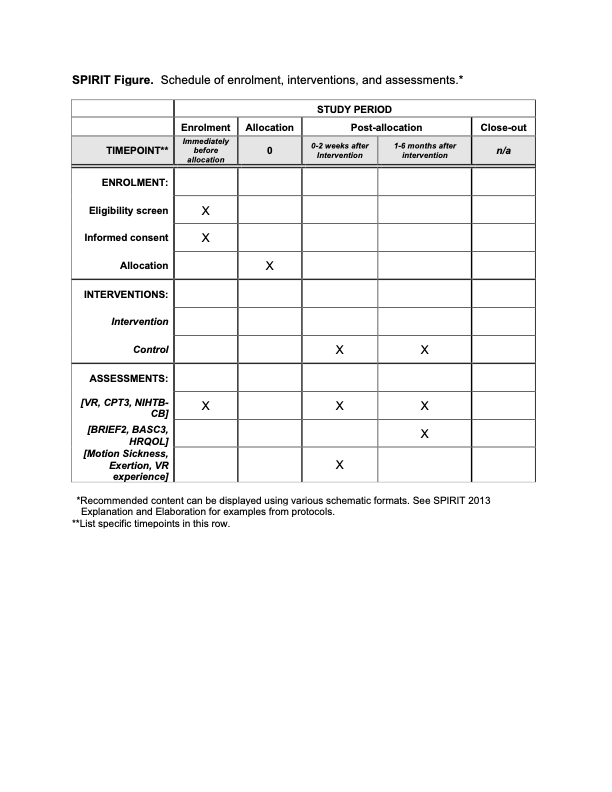

Supplement: Supplementary file 1 — Additional file 1. SPIRIT Figure. [file 13063_2024_8049_MOESM1_ESM.docx]
